# Assessing bed net damage: comparisons of three measurement methods for estimating the size, shape, and distribution of holes on bed nets

**DOI:** 10.1186/s12936-017-2049-8

**Published:** 2017-10-10

**Authors:** Jodi L. Vanden Eng, Don P. Mathanga, Keren Landman, Dyson Mwandama, Anna A. Minta, Monica Shah, James Sutcliffe, Joseph Chisaka, Kim A. Lindblade, Laura Steinhardt

**Affiliations:** 1 Malaria Branch, Division of Parasitic Diseases and Malaria, Centers for Disease Control and Prevention, 1600 Clifton Road, Atlanta, GA 30329 USA; 2 Malaria Alert Centre, University of Malawi College of Medicine, Blantyre, Malawi; 3 Epidemic Intelligence Service, Centers for Disease Control and Prevention, 1600 Clifton Road, Atlanta, GA 30329 USA; 4 Entomology Branch, Division of Parasitic Diseases and Malaria, Centers for Disease Control and Prevention, 1600 Clifton Road, Atlanta, GA 30329 USA

**Keywords:** Bed net, Physical durability, Hole index, Malaria, Vector control, Image analysis

## Abstract

**Background:**

Measuring the physical condition of long-lasting insecticidal nets (LLINs) under field conditions is of great importance for malaria control programmes to guide decisions on how frequently to replace LLINs. Current guidelines by the World Health Organization Pesticide Evaluation Scheme (WHOPES) propose a proportionate hole index (pHI) for assessing LLIN condition by counting the number of holes the size of a thumb, fist, head, and larger than a head. However, this method does not account for irregular hole shapes or exact hole sizes which could result in inaccurate decisions about when to replace LLINs.

**Methods:**

LLINs were collected during a 2013 health facility-based malaria case control study in Machinga District, Malawi. To evaluate the accuracy of the pHI, the physical condition of 277 LLINs was estimated by the WHOPES method and then compared with two more thorough measurement methods: image analysis of digital photographs of each LLIN side; and for 10 nets, ruler measurements of the length, width, and location of each hole. Total hole counts and areas per net were estimated by each method, and detailed results of hole shapes and composite pictures of hole locations were generated using image analysis.

**Results:**

The WHOPES method and image analysis resulted in similar estimates of total hole counts, each with a median of 10 (inter-quartile range (IQR) 4–24 and 4–23, respectively; p = 0.004); however, estimated hole areas were significantly larger using the WHOPES method (median 162 cm^2^, IQR 28–793) than image analysis (median 13 cm^2^, IQR 3–101; p < 0.0001). The WHOPES method classified fewer LLINs in ‘good condition’ compared to image analysis (42% vs 74%). The ruler method detected significantly more holes than image analysis did (p = 0.002) in 10 LLINs; however, total hole area was not significantly different (p = 0.16). Most holes were not circular but roughly 2–5 times longer in one direction. The lower quarter of LLIN sides was found to have the most holes.

**Conclusions:**

The WHOPES method overestimated total hole area, likely because holes are elongated rather than circular, suggesting further adjustments to the pHI formula may be warranted when considering LLIN replacement strategies.

**Electronic supplementary material:**

The online version of this article (doi:10.1186/s12936-017-2049-8) contains supplementary material, which is available to authorized users.

## Background

Insecticide-treated bed nets (ITNs) provide personal protection against malaria by forming a physical barrier against mosquitoes for people sleeping under the nets, while the insecticide deters and kills mosquitos coming in contact with the ITNs. In areas with stable malaria, high levels of ITN ownership and use can reduce the incidence of uncomplicated malaria by 50% and all-cause child mortality by 30% [1]. Hundreds of millions of ITNs, including long-lasting insecticidal nets (LLINs), have been distributed in malaria-endemic settings since 2004 as part of a multipronged approach to prevent and control malaria [2] and ITNs have been credited with having the biggest impact on malaria reductions since 2000 [3].

Over time, the protective qualities of LLINs will diminish as the level of insecticide drops or the net develops physical damage, such as seam rips, holes, or tears. To estimate when to replace nets, malaria control programmes monitor the physical condition of LLINs following guidelines published in 2011 by the World Health Organization Pesticide Evaluation Scheme (WHOPES) [4]. These guidelines provide a simple, standardized protocol to assess the physical condition of LLINs under field conditions by counting the number of holes that are approximately the size of a person’s thumb, fist, head, or larger than a head. The proportionate hole index (pHI), a weighted sum of the hole counts, is calculated for each net and is intended to provide an estimate of relative net damage. Based on the pHI, LLINs are further classified into categories of ‘serviceability’ such as ‘good’, ‘damaged’, and ‘too torn’ [5]. The pHI provides an easy method for evaluating overall net damage in field conditions but it ignores potentially useful and informative details about hole size, shape, and location on the net [6]. Other, more rigorous methods of measuring bed net holes, such as ruler-based measurements or image analysis, may provide further insight into the physical condition of LLINs.

Image analysis is a method typically used for analyzing fluorescence and bright-field microscopy data. This method processes a digital image to extract descriptive and statistical data about shapes and edges, object counts, area, and location. Image analysis has been used for several different malaria related applications unrelated to LLIN condition [7–12]. Image analysis has also been evaluated for the detection of defects in fabrics. In a study by Zhang, image analysis was used to process images of woven fabrics to detect and classify knots and slub (uneven thickening or lump) defects [13]. Drobina described the use of image analysis for the evaluation of the ends of spliced yarns [14]. To the researchers’ knowledge, nothing has previously been published on the use of image analysis for the evaluation of the physical condition of bed nets.

This study applies three methods of characterizing the physical condition of LLINs collected approximately 1 year following a mass distribution campaign in Machinga District, Malawi to estimate the accuracy of the pHI. The conventional method of surveyor-assessed holes using the WHOPES guidelines was compared to a novel application of image analysis of digital photographs of the LLINs as well as to detailed hand-calculated measurements using a ruler for a subset of 10 LLINs.

## Methods

### Study design

From March to September 2013, following a national distribution in July 2012 of Olyset^®^ Net LLINs (polyethylene nets manufactured by Sumitomo Chemical Co., Japan), a health facility based case–control study was conducted of children under 5 years of age presenting with malaria symptoms to the outpatient department of Machinga District Hospital in southern Malawi. The study aimed to assess the effectiveness of LLINs in an area of moderate pyrethroid resistance (for further details on methodology, please refer to Mathanga et al. [15]). The first 304 eligible children enrolled in the case–control study who were reported to sleep under the 2012 campaign net consistently (every night for 2 weeks before their illness onset), were asked to participate in the physical integrity sub-study. LLINs from these children were exchanged for new nets during a home visit and brought to the study clinic for further analysis.

### Bed net integrity assessments

#### Surveyor assessments using WHOPES criteria

**Fig. 1:**
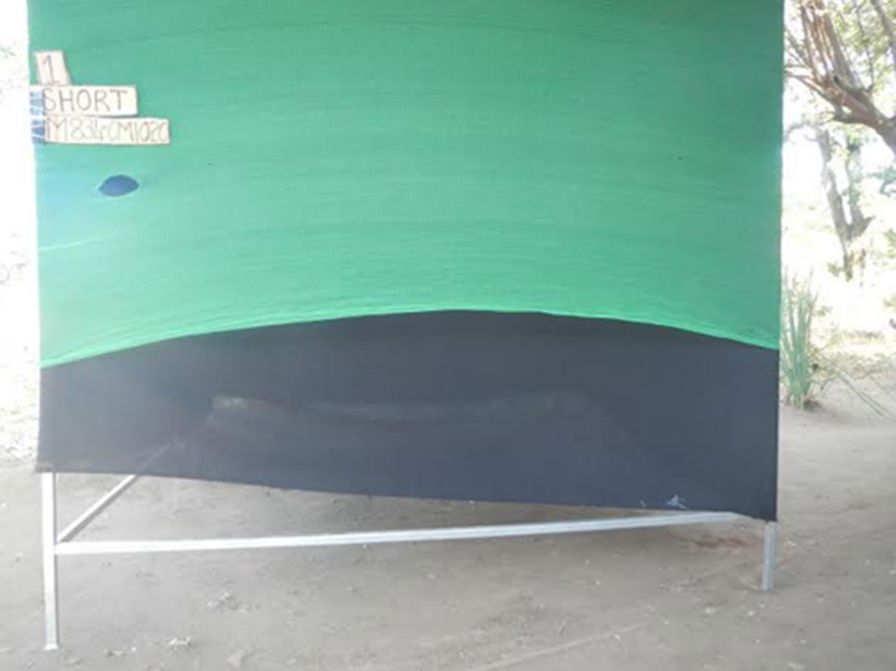
Photograph of a study net hung on a frame. Image of the bottom half of a short side of a study LLIN

At the study clinic, all LLINs collected during home visits were hung on a metal frame (dimensions: 180 × 190 × 150 cm) against which a black background was placed (Fig. 1). Surveyors analysed each side of the net and the roof and used a standard template [4] to count the number of holes in each of four size categories according to WHOPES criteria: (1) greater than 0.5 cm but smaller than a thumb (0.5 < diameter ≤ 2.0 cm), (2) larger than a thumb but smaller than a fist (2.0 < diameter ≤ 10.0 cm), (3) larger than a fist but smaller than a head (10.0 < diameter ≤ 25.0 cm), and (4) head-sized or larger (diameter > 25.0 cm). Holes judged to be 0.5 cm or less were not counted because the pHI method assumes mosquitoes cannot pass through holes of this size. Data were entered directly into handheld computers (Dell Axim X51, Round Rock, TX, USA) using questionnaires developed with Visual CE software (Syware, Inc., Cambridge, MA, USA). Data were downloaded directly into a Microsoft Access^®^ database (Redmond, WA, USA) for analysis.

#### Digital photographs

Surveyors photographed each side of the LLIN using a Nikon Digital Coolpix Camera (model number AW100V1.0, 16 Megapixels, Nikon Corporation, Tokyo, Japan). Surveyors stood approximately two metres from the net to capture images of the entire roof and the upper and lower half of each side and used standardized camera settings (compulsory flash, F-stop f/3.9, ISO-180, focal length 5 mm, metering mode: pattern, 35 mm focal length = 28, automatic white balance). Each photograph had a resolution of 4608 × 3456 pixels and was saved as a JPEG file. Prior to photographing, an identification marker was placed on each side of the LLIN with the study ID, the net side, and a reference scale. In some cases, different labels were used and additional adjustments had to be made to account for the non-standard labels. In addition, holes on the net that were not visible (small slits) and repairs were tagged with color coded tape for enhanced visibility in the image.

#### Image analysis of LLIN photographs

Images were analysed using ImageJ (http://rsb.info.nih.gov/ij/), a public domain, Java-based programme developed at the U.S. National Institutes of Health [16, 17]. Each LLIN took approximately 1 h on average to process using the image analysis software. ImageJ was used to identify the x and y coordinates for each hole by measuring the distance from the closest horizontal and vertical edge of the net side. The identification marker with the known reference scale was used to calibrate the unit of length to cm. Each hole was identified, increased in contrast, and converted to binary (black and white). The Analyze Particles tool was used to yield measurements of hole area (based on the black pixels representing the holes). Results were saved in a spreadsheet.

Image analysis provided measures in addition to hole area including: perimeter, circularity, and aspect ratio (AR). The perimeter was defined as the length of the outside boundary of the hole. Circularity was calculated as 4 × pi × (area/(perimeter^2^)). A circularity of 1 indicates a perfect circle, whereas values closer to 0 indicate an increasingly elongated shape. The aspect ratio is the ratio of the major axis to the minor axis assuming a fitted ellipse (AR = a/b). Holes of all sizes were measured and included in descriptions of hole shape and location. However, for consistency, only holes with a major axis ≥ 0.5 cm were included when comparing estimates of total hole counts or areas with the WHOPES and ruler methods. Due to uncertainty in the WHOPES measurement (the 0.5 cm cut-off was subjective, as exact measurements were not taken), an additional cut-off including holes ≥ 0.4 cm was also analysed to further examine the robustness of the WHOPES cut-off.

#### Ruler measurements

Subjective criteria were used in a non-exhaustive search to select a convenience sample of 10 nets with significant, but not extreme, amounts of damage (roughly 25–150 holes) for transport to Atlanta, GA. Study investigators (KL and JS) measured these nets using a ruler in the entomology laboratory at the U.S. Centers for Disease Control and Prevention (CDC). LLINs were placed over a frame made from PVC piping placed against a dark background. The length, width, and location (distance from the center of the hole to the bottom and side of the net noted as x and y coordinates) were measured using a tape measure (in mm). Holes < 0.5 cm were not counted.

### Data analysis

#### Hole area determinations

The area of individual holes on an LLIN was calculated separately for the three different assessment methods (Table 1). For the WHOPES method, the area recorded was the midpoint area of the hole size category based on the four WHOPES classifications (1.2, 28.3, 240.5, and 706.9 cm^2^, respectively) assuming the shape of the hole was a circle [4]. For the image analysis, area in cm^2^ (calibrated from square pixels) was provided directly from the ImageJ software output. Ruler measurements were converted to area using the formula for the area of an ellipse calculated as A = π × a × b where a is ½ the length of the major axis and b is ½ the length of the minor axis. Composite measures of damage for the entire LLIN were calculated by summing the areas for all holes on the net for each of the three assessment methods separately. The total hole surface area was then used to further classify LLINs into the following categories of ‘serviceability’: ‘good’ (< 79 cm^2^), ‘damaged’ (80–789 cm^2^) and ‘too torn’ (> 790 cm^2^) based on Table 1 in the Vector Control Technical Expert Group 2013 report [5].

**Table 1:** Summary of the three methods used to measure the physical condition of LLINs in this study

Method	Description	Determination of area
WHOPES	Field surveyors count the number of holes in each of four categories: 1. Less than thumb-sized but greater than 0.5 cm (0.5 < diameter ≤ 2.0 cm) 2. Larger than a thumb but smaller than a fist (2.0 < diameter ≤ 10.0 cm), 3. Larger than a fist but smaller than a head (10.0 < diameter ≤ 25.0 cm) 4. Head-sized or larger (diameter > 25.0 cm)	Each hole was assigned the midpoint area of the hole size category according to the guidelines: 1.2 cm^2^ 28.3 cm^2^ 240.5 cm^2^ 706.9 cm^2^ Note: no additional weighting was done
Image analysis	ImageJ software processed digital images of each LLIN side to extract statistical data about shapes and edges, object counts, area, and location	Analyse particles tool yielded area measurements in cm^2^ (calibrated from square pixels representing the holes)
Ruler	The length, width, and location (distance from the center of the hole to the bottom and side of the net noted as x and y coordinates) were measured using a tape measure (in mm)	The measured length and width were converted to area using the formula for the area of an ellipse calculated as A = π × a × b where a is ½ the length of the major axis and b is ½ the length of the minor axis

#### Statistical analyses

Data cleaning and analyses were performed using SAS^®^ version 9.32 (SAS Institute, Cary, NC, USA). Figures and plots were generated using ggplot in R [18] (version 3.3.1). This analysis included only LLINs with holes identified by one or more assessment method (91% of 277 LLINs). LLINs found to be intact by all three assessment methods were excluded from comparisons.

The nonparametric Wilcoxon signed rank test (paired difference) was used to compare paired measures of hole counts or areas for the same nets, such as the total area based on WHOPES categories compared to areas using image analysis. The Spearman correlation coefficient was used to measure the degree of linear association between the ranks of hole counts or total hole areas of LLINs between the different assessment methods. Data were presented visually using scatterplots with kernel density plots on the margins to show a smoothed distribution of total counts or damaged area by each method. Simple linear regression was used to test for association with normally distributed continuous outcomes. For other outcomes the Kruskal–Wallis test was used as a non-parametric method to compare hole measurements between methods using ranks. For hypothesis testing, holes from the same LLIN were assumed to be independent in terms of shape, size, and location. No adjustments were made for multiple testing. Bland and Altman plots (see Additional file 1: Figure S1) were used to provide graphical measure of agreement between measurement methods, where average differences close to zero are indicative of good agreement [19]; and log-transformed areas were used when differences between hole measurements were not normally distributed.

#### Visualization of composite hole damage

To better understand where most holes occur on LLINs, the image analysis measurements from all nets were combined to create a composite visual representation of hole damage. This was done separately for the short, long, and roof sides. All holes of all sizes (including holes < 0.5 cm in diameter) were plotted on an x–y coordinate system using ArcGIS (release 10.2, ESRI, Redlands, CA) and Quantum GIS [20] with a UTM zone projection which preserves distance. The plots used the packaged dimensions of the LLINs of 180 × 190 × 150 cm. Coordinates for the centroid of each hole were obtained from the image analysis which recorded hole locations as the distance from centroid of the hole to the left side of the net (x-coordinate) and to the bottom (lower half) or top (upper half) of the net (y-coordinate). This information, as well as the area and the AR were used to derive the length of the major and minor axes for each hole assuming the holes formed perfect ellipses (no irregularly shaped edges). Elliptical holes were assumed to orient horizontally as no angular information was available. Holes from each net side were represented as different point layers. The point layers were first converted to ellipses as polygon shapefiles and then raster grids (cell size = 0.01). Spatial analyst tools were used to sum across raster layers (net sides) to create the final composite image.

The centroids of the holes were used to generate hot spot ‘maps’ which identify grid areas on the LLIN side with statistically significant higher (red) or lower (blue) than expected counts (incidence) of holes on the LLIN side compared to a random process. Maps were generated using the optimized hot spot analysis tool in the spatial statistics toolbox in ArcGIS and the resulting map is based on the Getis-Ord Gi* statistic [21].

## Results

### Bed net holes summary

A total of 277 LLINs were collected from the 304 eligible children and assessed by surveyors in the field for holes following the WHOPES method. Image analysis data were available for 258 of the 277 LLINs and ruler measurements were performed on 10 LLINs. Twenty-four LLINs had no holes identified by any method; these nets were excluded from further analysis. Overall, 234 LLINs with holes had both WHOPES counts and image analysis performed on them, and ten of these had ruler measurements as well.

The WHOPES method classified 66% of holes as ‘smaller than a thumb’, 27% ‘between thumb and fist’, 5% ‘between fist and head’, and 2% ‘larger than a head’. For comparison purposes, holes from the image analysis were put into WHOPES size categories based on the estimated areas from the image analysis. Overall, 84% of holes were classified as ‘smaller than a thumb’, 13% ‘between thumb and fist’, 2% ‘between fist and head’, and < 0.3% ‘larger than a head’ for the image analysis.

### Comparison of hole counts

Among 234 LLINs with holes, the WHOPES method identified 4863 holes whereas the image analysis method identified 4415 holes. Both methods yielded the same median number of holes per LLIN of 10 holes and similar inter-quartile range (IQR) of 4–24 holes (WHOPES) and 4–23 holes (image) (Table 2). The distribution of the median number of holes per LLIN was significantly different between the WHOPES and image analysis methods (Wilcoxon signed rank, p = 0.004). When the cut-off was changed to exclude holes with diameters < 0.4 cm (as opposed to 0.5 cm) for the image analysis this difference was no longer significant (see Additional file 2: Table S1, p = 0.11).

**Table 2:** Descriptive statistics of total hole counts and total hole areas as measured using WHOPES guidelines and image analysis

Statistic	Total hole counts		Total area (cm^2^)	
	WHOPES	Image	WHOPES	Image
Minimum	0	0	0	0
1st quartile	4	4	28	3
Median	10	10	162	13
Mean	21	19	778	187
3rd quartile	24	23	793	101
Maximum	359	248	12,840	2930
Total	4863	4415		

Restricted to holes with diameters ≥ 0.5 cm. N = 234 LLINs

*WHOPES* World Health Organization Pesticide Evaluation Scheme

**Fig. 2:**
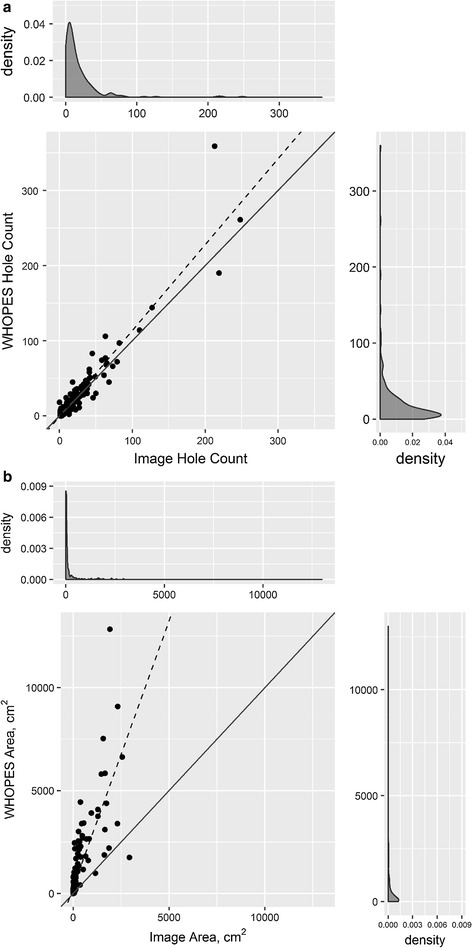
Total hole counts (**a**) and total hole area (**b**) as measured using WHOPES assessment and image analysis methods. Scatterplot displaying the **a** number of holes or **b** total area of hole damage in each LLIN (represented by a dot) as measured by the WHOPES methodology (y-axis) and image analysis (x-axis). The dashed line represents a fitted line assuming a simple linear regression model. The solid diagonal line represents y = x. Kernel density plots on the margins show a smoothed distribution of counts (or area) by each method

A scatterplot displaying the total hole counts per LLIN measured by the WHOPES method and image analysis method is shown in Fig. 2a. LLINs with the exact same hole counts by both methods lie along the solid diagonal line (n = 31 of 234, 13%); the slope of 1.1 indicates slightly greater hole counts by the WHOPES method compared to the image analysis method (intercept = − 0.8). The two methods have a Spearman’s correlation coefficient of ρ = 0.93 (p < 0.0001).

For the 10 LLINs measured with a ruler, the WHOPES hole counting method identified 468 holes, the image analysis 444, and the ruler method 602 holes (Table 3). The medians (IQR) were 34 (26–54), 34.5 (24–58), and 41 (37–72); respectively. The ruler method yielded significantly larger hole counts than image analysis (Wilcoxon signed rank p < 0.002), and the Spearman’s correlation coefficient between the two methods was ρ = 0.84 (p = 0.003).

**Table 3:** Descriptive statistics of total hole counts and total hole area as measured using WHOPES guidelines, ruler measurements, and image analysis

Statistic	Total hole counts			Total area (cm^2^)		
	WHOPES	Image	Ruler	WHOPES	Image	Ruler
Minimum	25	21	27	63	10	21
1st quartile	26	24	37	379	70	111
Median	34	34.5	41	2457	576	629
Mean	47	44	60	3486	797	1127
3rd quartile	54	58	72	4380	1470	1815
Maximum	114	110	142	12,839	1919	4301
Sum	468	444	602	34,862	7965	11,269

Restricted to holes with diameters ≥ 0.5 cm. The World Health Organization Pesticide Evaluation Scheme. n = 10 LLINs

### Comparison of hole areas

The WHOPES method estimated a median hole area per LLIN of 162 cm^2^ (IQR 28–793, maximum: 12,840 cm^2^), whereas the image analysis estimated a median hole area of 13 cm^2^ (IQR 3–101, maximum: 2930 cm^2^) (Table 2). The WHOPES method had an average area of hole damage per LLIN of 778 cm^2^ compared to an average of 187 cm^2^ by the image analysis (Wilcoxon signed rank p < 0.0001). When the cut-off was expanded to include holes with diameters between 0.4 and 0.5 cm for the image analysis this difference remained significant (p < 0.0001).

Using the VCTEG-suggested categorizations of ‘serviceable’ nets based on total holed surface area, results from the WHOPES method found 42% of the 234 study nets were in ‘good condition’, 33% ‘damaged’, and 25% ‘too torn’. In contrast, using areas calculated by image analysis, 74% of the study LLINs in ‘good condition’, 19% ‘damaged’, and 7% ‘too’ torn based on the same categorizations.

A scatterplot displaying the total hole area per LLIN measured by the WHOPES method and image analysis method is shown in Fig. 2b. The simple linear regression model had a slope of 2.6 (intercept = 295), indicating that on average the WHOPES-measured total hole areas were more than twice that of the image analysis-calculated areas. The two methods have a Spearman’s correlation coefficient of ρ = 0.90 (p < 0.0001).

For the 10 LLINs with ruler measurements, median hole areas were 2457 cm^2^ (IQR 379–4380), 576 cm^2^ (IQR 70–1470), and 629 cm^2^ (IQR 111–1815); for the WHOPES, image analysis, and ruler methods, respectively (Table 3). No significant difference in the estimated area damaged by holes was found between the ruler and image analysis methods (Wilcoxon signed rank p = 0.16). The two methods have a Spearman’s correlation coefficient of ρ = 0.94 (p < 0.0001).

### Hole shape characteristics

**Fig. 3:**
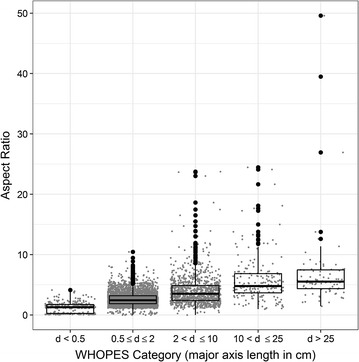
Aspect ratio of holes measured using image analysis. Jitter plot with box and whisker plot overlay displaying the distribution of the aspect ratio of holes as measured by image analysis (shown separately for the different WHOPES hole size categories, d = diameter). The jitter plots show the distribution of aspect ratio (each hole is represented by a dot). The box in the box and whisker plot show the 75th percentile, median, and 25th percentile, respectively; whereas the whiskers identify the extremes, including the minimum and maximum. Aspect ratio is the ratio of the major axis to the minor axis

The image analysis measured the aspect ratio, defined as the ratio of the major axis to the minor axis. Results indicated that in general holes were more than twice as long as they were wide (median AR = 2.6, IQR = 1.9–3.7, mean = 3.1, Table 4). Larger AR values were significantly associated with larger size categories (Kruskal–Wallis p < 0.0001) indicating that in general larger holes were more elongated than smaller holes. The median AR was 5.5 (IQR 4.3–7.5) for holes with diameter > 25.0 cm; whereas small holes with a diameter between 0.5 and 2 cm had a median AR of 2.4 (IQR 1.9–3.2). Box and whisker plots display the distribution of aspect ratios classified by WHOPES categories (Fig. 3). Analogously, circularity decreased as hole size increased (Table 4, Additional file 3: Figure S2).

**Table 4:** Descriptive statistics of hole shape using image analysis

Size group^a^ (diameter in cm)	N	Circularity		Aspect ratio		
		Mean	Std. dev.	Median	25th percentile	75th percentile
Smaller than 0.5 cm < 0.5 cm^b^	274	0.53	0.28	1.3	0.25	1.7
Smaller than thumb (0.5 ≤ diameter ≤ 2.0 cm)	3147	0.46	0.16	2.4	1.9	3.2
Larger than thumb, smaller than fist (2.0 < diameter ≤ 10.0 cm)	1005	0.35	0.14	3.5	2.4	4.9
Larger than fist, smaller than head (10.0 < diameter ≤ 25.0 cm)	180	0.28	0.14	4.8	3.7	6.9
Larger than head (diameter > 25.0 cm)	83	0.25	0.15	5.5	4.3	7.5
Total (all holes)	4689	0.43	0.18	2.6	1.9	3.7

Circularity indicates the amount of elongation of the hole (1 indicates a perfect circle, values closer to 0 indicate more elongation). Aspect ratio is the ratio of the major axis to the minor axis

^a^Diameter was approximated as the length of the major axis

^b^Not measured with the World Health Organization Pesticide Evaluation Scheme (WHOPES) guidelines

### Hole location

**Fig. 4:**
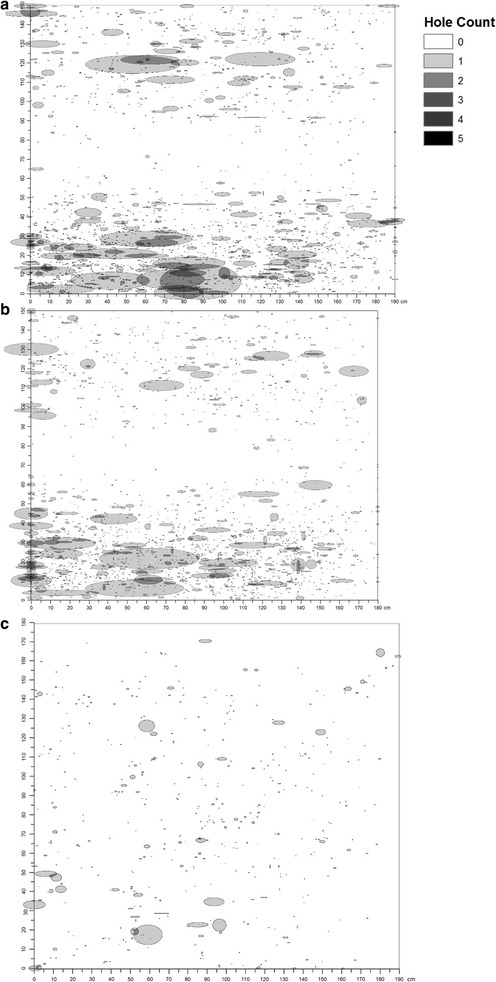
Composite visualization of hole damage by net side. Composite visualization of hole damage for the **a** long, **b** short, and **c** roof sides of study LLINs. Holes (represented as ellipses) are plotted based on measurements from image analysis (centroid, area and aspect ratio) for each net side, and sides of the same type were overlaid to form a composite image

**Fig. 5:**
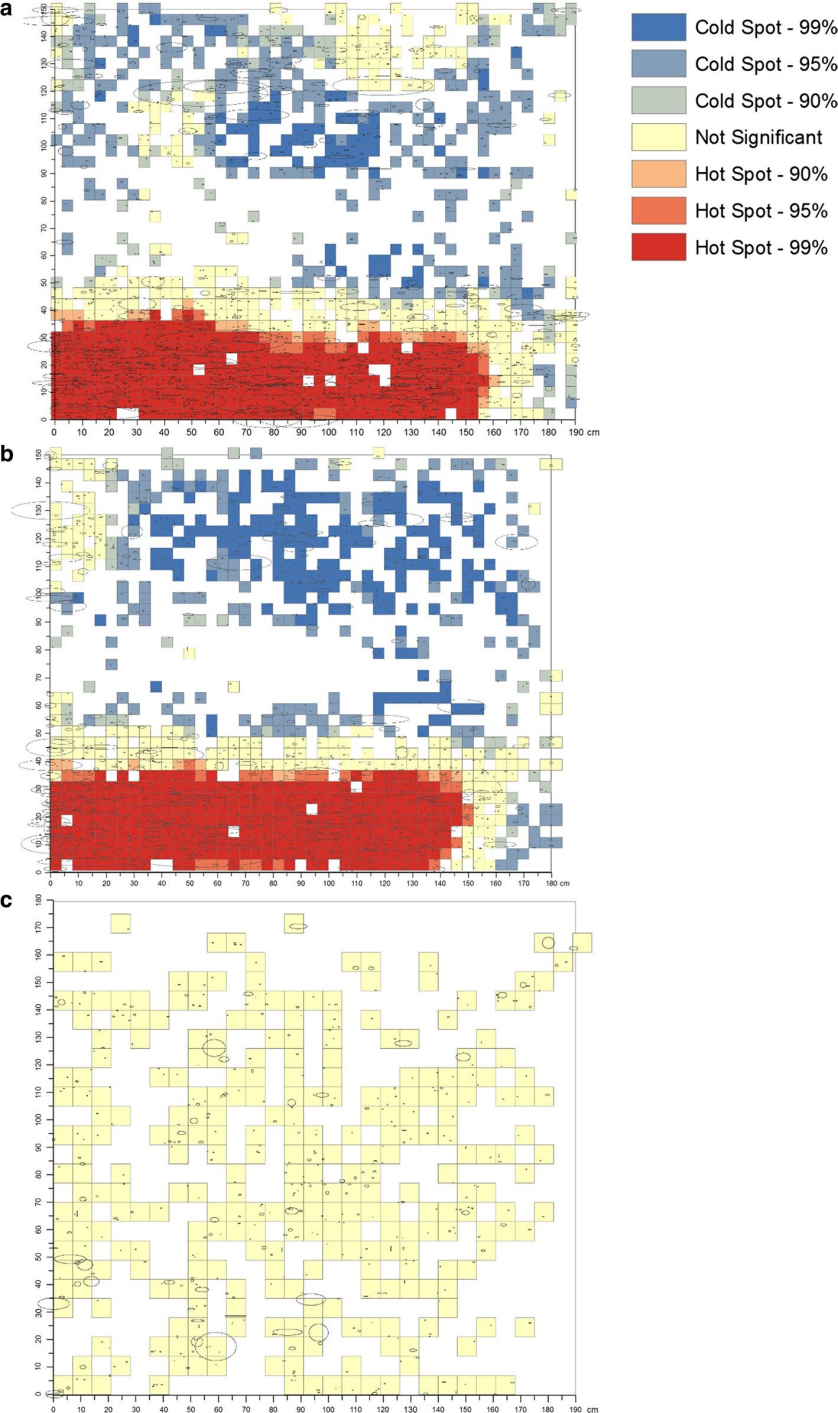
Hotspot map of hole damage by net side. Hot spot analysis of the composite images of hole damage using the Getis-Ord Gi* statistic for the **a** long, **b** short, and **c** roof sides of study LLINs. This statistic identifies areas (grids) on the LLIN side with statistically significant higher (red) or lower (blue) than expected counts (incidence) of holes on the LLIN side compared to a random process

Based on the image analysis measurements, holes of all sizes from all nets were combined to create a composite visual representation of hole damage after 1 year of use for the short, long, and roof sides of the LLINs (Figs. 4a–c). Significant hot spots were found on the lower quarter (30–40 cm) of nets on the short and long sides indicating locations where high hole counts are clustered (Figs. 5a–c). Significant cold spots were identified on portions of the middle and upper sections of the short and long sides which reflects spatial clusters of grids with very low hole counts. No significant hot or cold spots of hole counts were found on the roof section.

## Discussion

The physical durability of LLINs is of great interest to malaria control programmes to better estimate the most cost effective timing and distribution of replacement nets. In this study a novel application of image analysis techniques was used to assess the physical condition of LLINs in a standardized, objective, quantifiable, and reproducible manner. These techniques allowed us to obtain more detailed information about the sizes, shapes, and distributions of holes on LLINs, and to compare these results to the standard field method outlined by WHOPES guidelines. This information may be useful for understanding patterns of degradation and better refining the estimate of a ‘useful life’ of an LLIN.

This study found that after approximately 1 year of use, most LLINs were in ‘good’ condition (74%) according to the VCTEG-suggested categorizations of ‘serviceable’ nets using results from the image analysis. There were not many holes (median number of holes per net was 10); and most (85%) of the holes were smaller than a thumb in size.

Hole counting via image analysis resulted in similar counts to the WHOPES method; however, total estimated hole area from the WHOPES method was significantly larger than from the image analysis. This resulted in far more WHOPES-scored nets classified as ‘damaged’ and ‘too torn’ (58%) compared to the image analysis (26%). One reason for this may be that the WHOPES method assumes all holes are circular. This overlooks the possibility of irregularly shaped holes. Moreover, a wide range of holes of different sizes are assigned the same area because they fall into the same broad size category of ‘thumb’, ‘fist’, and ‘head’ rather than being assigned their actual area. A study by Vanden Eng et al. [6] found this tended to overestimate the actual hole area for small, medium, and large holes and underestimate it for extra-large holes.

Image analysis data showed that most LLIN holes were not circular as assumed by the WHOPES method but elongated. Median aspect ratios ranged from 2.3 for holes smaller than a thumb to 5.2 for holes larger than a head. This means that most holes were roughly 2–5 times longer in one direction than the other, and as a result, assuming the hole is circular tends to overestimate the actual holed area by roughly 2–5 times. The ARs determined using image analysis in this study were similar to those approximated for polyethylene LLINs in a net durability study in Mozambique [6]. In that study the mean AR for all holes was 2.4 (small 1.9, medium 2.6, large 4.2, and extra-large 6.6). In the current study the mean AR for all holes was 3.2. It may be possible, based on weave patterns of various brands, to develop a formula or set of standard weights that could better adjust the WHOPES pHI for the elongated shape of holes. This will not correct for all of the limitations of the pHI (such as the pooling of holes into broad size categories), though it may help prevent prematurely assigning LLINs to a ‘damaged’ or ‘too torn’ classification and better reflect the true lifespan of the LLINs.

For the 10 LLINs with ruler measurements, hole counting appeared to identify significantly more holes than image analysis, but the total estimated hole area was not significantly different from image analysis estimates. It is possible the image analysis missed some holes due to poor contrast or image quality, while the ruler method calculated area using the formula for an ellipse, not a circle, perhaps providing a closer estimate of area.

Understanding what parts of nets are most prone to holes can help inform efforts to strengthen these locations during the manufacturing process. This study identified the bottom quarter of the LLIN sides to have the highest hole counts. This section may be more susceptible to tears based on use or other factors. For example, it is possible this section is tucked under a mattress or more exposed to animals or objects catching and snagging the LLIN [22]. When determining the overall ‘serviceability’ of an LLIN, future guidelines may want to take into account not only the size of the hole, but also the location. Sutcliffe and Yin showed that many times more *Anopheles gambiae* attack the lower one-third of a net than the upper two-thirds [23]. Their findings also showed the roof is attacked about 30 times more intensely than the lower sides, meaning that even small holes on the roof may represent much more mosquito entry risk than larger holes elsewhere on the bed net. Holes in locations on a net that may be associated with higher mosquito penetration and malaria risk could possibly be assigned different weights or values during assessments of physical condition. Moreover, manufacturers can use this information to build stronger LLINs. Some manufacturers are already providing cotton sheet roofs and reinforced bottom borders to help prevent tearing when tucked underneath a mattress.

Image analysis techniques allowed a detailed investigation of the physical degradation patterns of LLINs and a comparison with the WHOPES method. Nevertheless, the image analysis requires several image processing steps and is not yet practical to carry out in the field. A more efficient tool, such as a portable, field-ready smartphone application, would need to be created before this type of detailed analysis could be feasibly performed in a routine LLIN durability assessment. Image analysis and ruler measurements may yield more accurate area estimates than the WHOPES method, but are more labour-intensive. Simpler methods, such as the current WHOPES method, are more logistically feasible, but at a cost of accuracy due to measurement errors.

In most cases, bed net durability assessments are cumbersome and time consuming; and even the simpler WHOPES method involves counting all of the holes on selected LLINs. As a result, these assessments are generally performed on a small, non-representative sample of LLINs and findings should be interpreted with caution when extrapolating to the general population or attempting to make programmatic decisions of net replacement at the national level. Efforts carrying out assessments using a larger sample of LLINs that is representative of the population are possible through routine Malaria Indicator Surveys (MIS) or Demographic Health Surveys; but require a simple and rapid measure of hole damage (e.g. at least one hole larger than a fist) to be accepted. As an example, a 2015 MIS in Kenya asked about holes in each bed net and recorded “no holes” or the size of the largest hole for each net [24]. Population-based surveys would allow informed decisions on when to replace LLINs; moreover, the MIS data provides the possibility to explore potential associations with malaria prevalence and bed net durability. The durability measure remains elusive; however, in part because the exact relationship between the physical condition of LLINs and the corresponding increased epidemiological risk of malaria, has yet to be elucidated.

There are several limitations of the current study that should be noted. Pictures of the LLINs were taken outdoors, and image quality due to lighting or other factors may have impacted the data from the image analysis. LLINs were placed over a frame which may have caused the holes to stretch or elongate, thereby introducing measurement error. LLINs may also have shrunk under field conditions whereas the composite image assumed the original net dimensions of (180 × 190 × 150 cm). Since the location of the holes were measured as the distance from the left edge (x-axis) or top or bottom edge (y-axis), net shrinkage may generate a ‘gap’ on the right or center of the composite images for nets in which the dimensions assumed in the plots are greater than the actual shrunken dimensions of the LLIN. Only 10 non-randomly selected LLINs were measured with a ruler, so a complete comparison among the three methods could not be made. Lastly, when generating a composite visualization of net damage, all holes were assumed to be horizontal and shaped as ellipses.

## Conclusions

Results from this study indicate that both the WHOPES and image analysis methods identify and count holes similarly; however, the WHOPES method overestimates total hole area. One possible explanation is because it assumes net holes are circular, while image analysis showed holes were more commonly elongated or elliptical. Although more accurate, current image analysis methods for characterizing LLIN holes were time- and labour-intensive making it challenging for use as a practical field tool. The current WHOPES method with its simple field implementation might be sufficient for categorizing net physical damage, but further adjustments to improve accuracy could be considered that assume elliptical, rather than circular, holes. Moreover, efforts to identify even simpler measurement tools to enable durability testing on larger population representative samples of LLINs may better inform malaria programme replacement strategies.

## Additional files

**Additional file 1: Figure S1.** Bland and Altman Plots of total hole counts (A) and total hole area (B) as measured using WHOPES assessment and image analysis methods.

**Additional file 2: Table S1.** Descriptive statistics of total hole counts and total hole areas as measured using WHOPES guidelines and image analysis*. n = 234 LLINs.

**Additional file 3: Figure S2.** Circularity of holes measured using image analysis.

## References

[1] Lengeler C (2004). Insecticide-treated bed nets and curtains for preventing malaria. Cochrane Database Syst Rev..

[2] WHO. World Malaria Report. Geneva: World Health Organization; 2013.

[3] Bhatt S, Weiss DJ, Cameron E, Bisanzio D, Mappin B, Dalrymple U (2015). The effect of malaria control on *Plasmodium falciparum* in Africa between 2000 and 2015. Nature.

[4] WHO. Guidelines for monitoring the durability of long-lasting insecticidal mosquito nets under operational conditions. Geneva: WHO/HTM/NTD/WHOPES; 2011.

[5] WHO. Vector Control Technical Expert Group Report to Malaria Policy and Advisory Committee Meeting. vol. Session 6.1. Geneva: World Heatlh Organization; 2013.

[6] Vanden Eng JL, Chan A, Abilio AP, Wolkon A, Ponce de Leon G, Gimnig J (2015). Bed net durability assessments: exploring a composite measure of net damage. PLoS ONE.

[7] Gering E, Atkinson CT (2004). A rapid method for counting nucleated erythrocytes on stained blood smears by digital image analysis. J Parasitology.

[8] Frean JA (2009). Reliable enumeration of malaria parasites in thick blood films using digital image analysis. Malar J..

[9] Chambers EW, Bossin HC, Ritchie SA, Russell RC, Dobson SL (2013). Landing response of Aedes (Stegomyia) polynesiensis mosquitoes to coloured targets. Med Vet Entomol.

[10] Mains JW, Mercer DR, Dobson SL (2008). Digital image analysis to estimate numbers of Aedes eggs oviposited in containers. J Am Mosq Control Assoc.

[11] Delves MJ, Sinden RE (2010). A semi-automated method for counting fluorescent malaria oocysts increases the throughput of transmission blocking studies. Malar J.

[12] Hoffmann WC, Jank PC, Klun JA, Fritz BK (2010). Quantifying the movement of multiple insects using an optical insect counter. J Am Mosq Control Assoc.

[13] Zhang YXF, Bresee RR (1995). Fabric defect detection and classification using image-analysis. Text Res J.

[14] Drobina R, Machnio MS (2006). Application of the image analysis technique for estimating the dimensions of spliced connections of yarn-ends. Fibres Text East Eur.

[15] Mathanga DP, Mwandama DA, Bauleni A, Chisaka J, Shah MP, Landman KZ (2015). The effectiveness of long-lasting, insecticide-treated nets in a setting of pyrethroid resistance: a case-control study among febrile children 6 to 59 months of age in Machinga District, Malawi. Malar J..

[16] Schneider CA, Rasband WS, Eliceiri KW (2012). NIH Image to ImageJ: 25 years of image analysis. Nat Methods.

[17] Hartig SM. Basic image analysis and manipulation in ImageJ. Curr Protoc Mol Biol. 2013;Chapter 14:Unit 14.15. 10.1002/0471142727.mb1415s102 23547012

[18] R Core Team. R: A language and environment for statistical computing. In: R Foundation for Statistical Computing. Vienna, Austria. https://www.R-project.org/2016.

[19] Bland JM, Altman DG (1986). Statistical methods for assessing agreement between two methods of clinical measurement. Lancet.

[20] QGIS Development Team. QGIS Geographical Information System. In: Open Source Geospatial Foundation Project. http://www.qgis.org/2016.

[21] Getis A, Ord JK (1992). The analysis of spatial association by use of distance statistics. Geogr Anal..

[22] Tan KR, Coleman J, Smith B, Hamainza B, Katebe-Sakala C, Kean C (2016). A longitudinal study of the durability of long-lasting insecticidal nets in Zambia. Malar J..

[23] Sutcliffe JF, Yin S (2014). Behavioural responses of females of two anopheline mosquito species to human-occupied, insecticide-treated and untreated bed nets. Malar J..

[24] National Malaria Control Programme (NMCP), Kenya National Bureau of Statistics (KNBS), and ICF International. 2016. Kenya Malaria Indicator Survey 2015. Nairobi: NMCP, KNBS, and ICF International.

