# Supplementary material for: Assessing bed net damage: comparisons of three measurement methods for estimating the size, shape, and distribution of holes on bed nets
Source: Malar J. 2017 Oct 10;16:405. doi: 10.1186/s12936-017-2049-8 (PMC5635507; doi:10.1186/s12936-017-2049-8)
Supplement: Supplementary file 1 — Additional file 1: Figure S1. Bland and Altman Plots of total hole counts (A) and total hole area (B) as measured using WHOPES assessment and image analysis methods. [file 12936_2017_2049_MOESM1_ESM.docx]

**A**


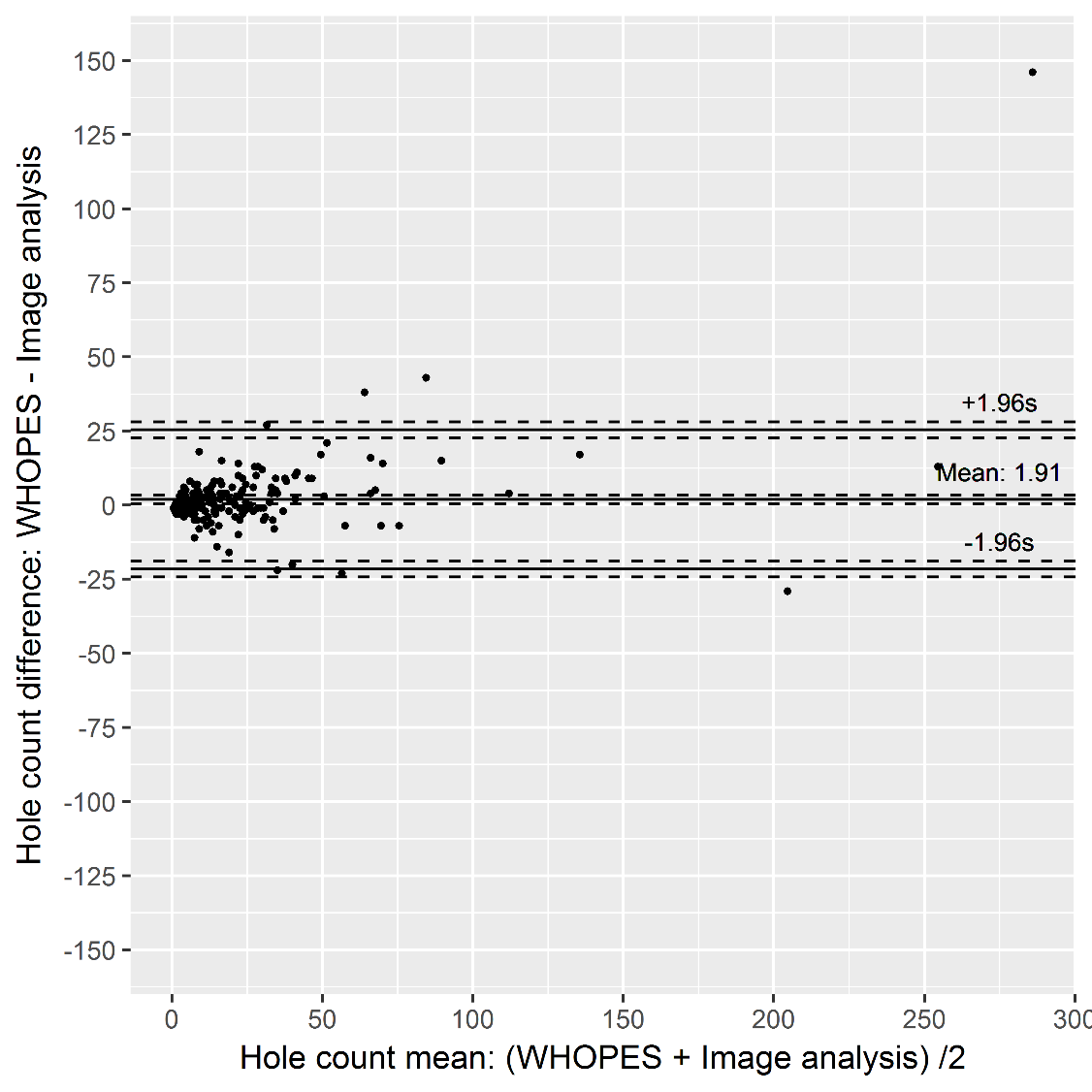

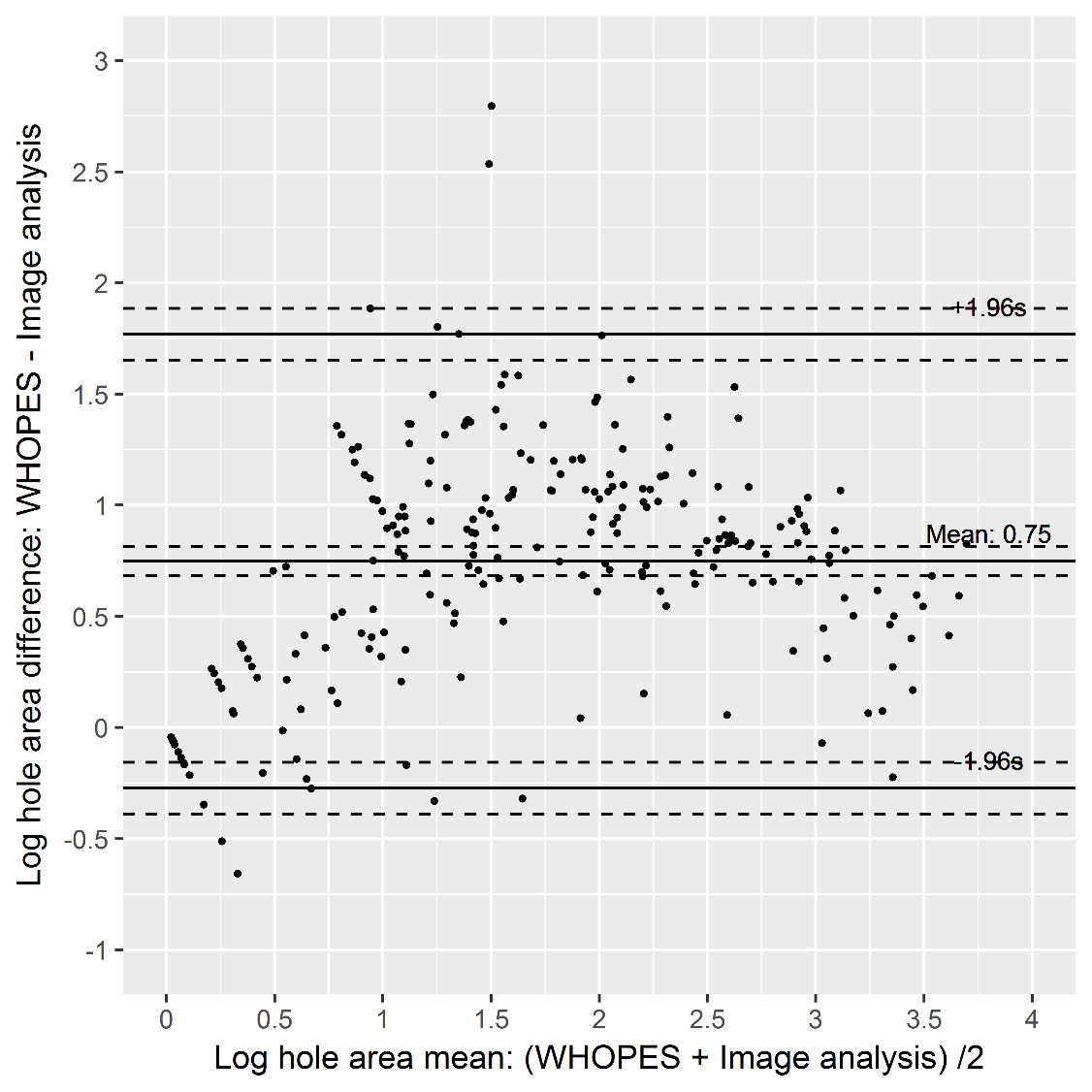


**B**

## **Figure S1: Bland and Altman Plots of total hole counts (A) and total hole area (B) as measured using WHOPES assessment and image analysis methods**

Bland and Altman plots displaying the (A) number of holes or (B) log total hole area as measured by the WHOPES methodology and image analysis. Differences (y-axis) are plotted against the means (x-axis). The solid horizontal lines represent the mean difference and upper and lower 95% limits of agreement. The dashed lines represent the 95% confidence intervals on these values (as a measure of precision).

Bland Altman plots provide an alternative approach to assessing agreement between different measures based on graphical techniques. Bland and Altman plot (Fig. S1A) shows the average difference in hole counts between assessment methods is 1.9 (95% CI: -0.4, 3.5) holes with a lower limit of agreement of -21.6 (95% CI: -18.9, -24.3), and an upper limit of agreement of 25.4 (95% CI: 22.7, 28.1). A Bland and Altman plot (Fig. S1B) shows the average difference in estimated area between assessment methods using log-transformed areas. The mean difference on the log scale is 0.74 (95% CI: 0.68, 0.82) with a lower limit of agreement of -0.27 (95% CI: -0.39, -0.16), and an upper limit of agreement of 1.77 (95% CI: 1.65, 1.88). Taking the antilog of the limits gets 0.53 and 57.6. Bland and Altman analysis indicated that the WHOPES measures of estimated area differ from the image analysis measure by 46% below to 588% above, based on a 95% confidence level.
