# Supplementary material for: Assessing bed net damage: comparisons of three measurement methods for estimating the size, shape, and distribution of holes on bed nets
Source: Malar J. 2017 Oct 10;16:405. doi: 10.1186/s12936-017-2049-8 (PMC5635507; doi:10.1186/s12936-017-2049-8)
Supplement: Supplementary file 2 — Additional file 2: Table S1. Descriptive statistics of total hole counts and total hole areas as measured using WHOPES guidelines and image analysis*. n = 234 LLINs. [file 12936_2017_2049_MOESM2_ESM.docx]

## Table S1. Descriptive statistics of total hole counts and total hole areas as measured using WHOPES guidelines and image analysis*. n = 234 LLINs.

|  | **Total Hole Counts** | | **Total Area (cm^2^)** | |
| --- | --- | --- | --- | --- |
| **Statistic** | **WHOPES** | **Image *** | **WHOPES** | **Image *** |
| Min | 0 | 0 | 0 | 0 |
| 1^st^ Quartile | 4 | 4 | 28 | 3 |
| Median | 10 | 10 | 162 | 13 |
| Mean | 21 | 19 | 778 | 187 |
| 3^rd^ Quartile | 24 | 23 | 793 | 101 |
| Max | 359 | 251 | 12,840 | 2,930 |
| Total | 4,863 | 4,559 |  |  |

* Image analysis expanded cut-off to include holes with diameters between 0.4 and 0.5 cm.
