# Supplementary material for: Assessing bed net damage: comparisons of three measurement methods for estimating the size, shape, and distribution of holes on bed nets
Source: Malar J. 2017 Oct 10;16:405. doi: 10.1186/s12936-017-2049-8 (PMC5635507; doi:10.1186/s12936-017-2049-8)
Supplement: Supplementary file 3 — Additional file 3: Figure S2. Circularity of holes measured using image analysis. [file 12936_2017_2049_MOESM3_ESM.docx]

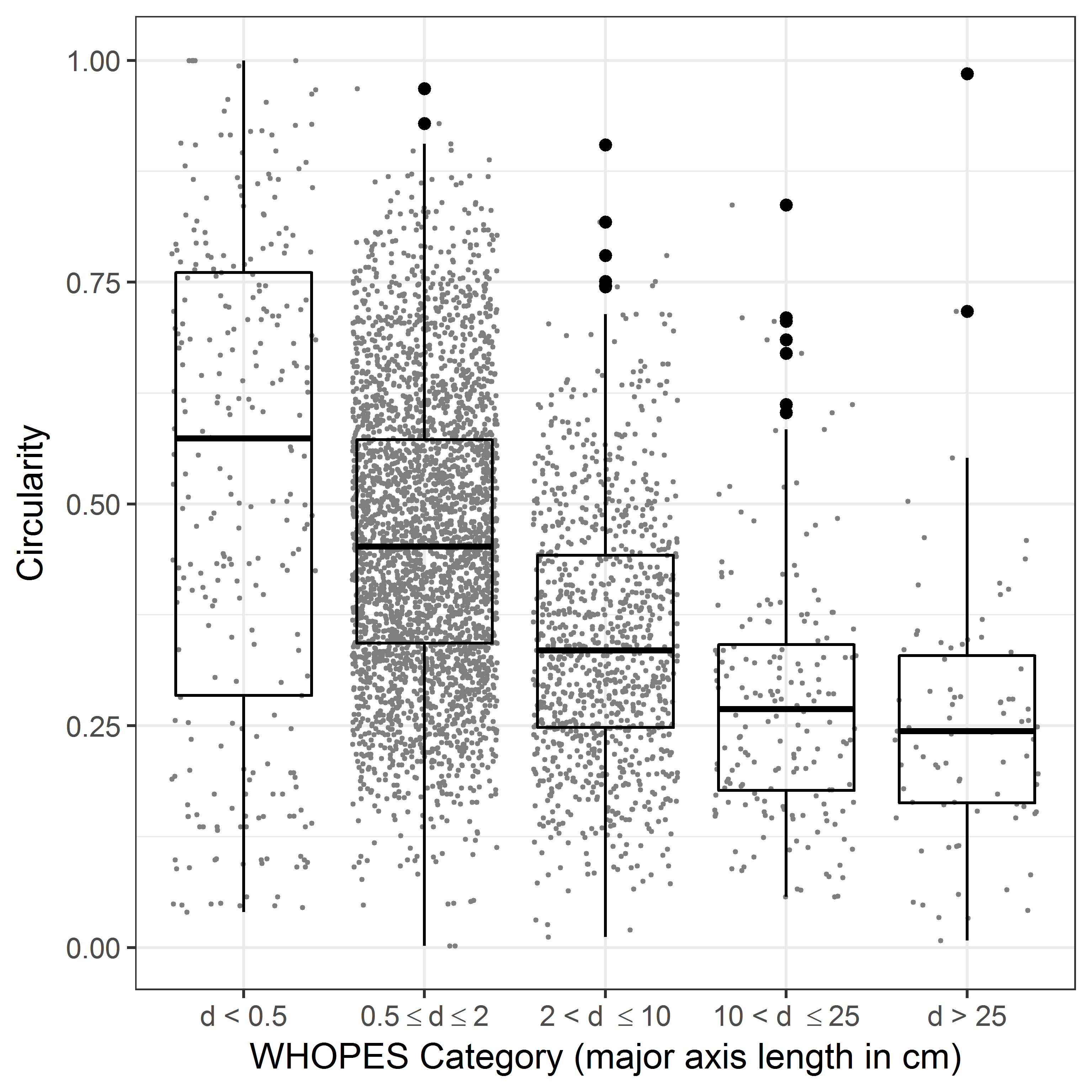


Figure S2: Circularity of holes measured using image analysis.

Jitter plot with box and whisker plot overlay displaying the distribution of the circularity of holes as measured by image analysis (shown separately for the different WHOPES hole size categories, d = diameter). The jitter plots show the distribution of circularity (each hole is represented by a dot). The box in the box and whisker plot show the 75^th^ percentile, median, and 25^th^ percentile, respectively; whereas the whiskers identify the extremes, including the minimum and maximum.

Circularity indicates the amount of elongation of the hole (1 indicates a perfect circle, values closer to 0 indicate more elongation). The average circularity of all holes was 0.43 (standard deviation = 0.18). Larger holes tended to be less circular than smaller holes (mean circularity = 0.25 for holes with diameter > 25.0 cm; and circularity = 0.46 for holes with a diameter between 0.5 and 2 cm; Table 4). Circularity was significantly associated with hole area (linear regression p-value < 0.0001), with a 0.002 unit decrease in circularity for every 10 cm^2^ increase in area.
